# The impact of extended bed rest on the musculoskeletal system in the critical care environment

**DOI:** 10.1186/s13728-015-0036-7

**Published:** 2015-10-09

**Authors:** Selina M. Parry, Zudin A. Puthucheary

**Affiliations:** Department of Physiotherapy, School of Health Sciences, The University of Melbourne, Level 7 Alan Gilbert Building, Parkville, Melbourne, VIC 3010 Australia; Division of Respiratory and Critical Care Medicine, National University Health System, Singapore, Singapore; Institute of Health and Human Performance, University College London, London, UK

**Keywords:** Critical illness, Muscle wasting, Intensive care unit-acquired weakness, Sepsis, Muscle protein turnover, Rehabilitation, Bed rest

## Abstract

Prolonged immobility is harmful with rapid reductions in muscle mass, bone mineral density and impairment in other body systems evident within the first week of bed rest which is further exacerbated in individuals with critical illness. Our understanding of the aetiology and secondary consequences of prolonged immobilization in the critically ill is improving with recent and ongoing research to establish the cause, effect, and best treatment options. This review aims to describe the current literature on bed rest models for examining immobilization-induced changes in the musculoskeletal system and pathophysiology of immobilisation in critical illness including examination of intracellular signalling processes involved. Finally, the review examines the current barriers to early activity and mobilization and potential rehabilitation strategies, which are being, investigated which may reverse the effects of prolonged bed rest. Addressing the deleterious effects of immobilization is a major step in treatment and prevention of the public health issue, that is, critical illness survivorship.

## Background

Advances in medical knowledge and technology have led to improvements in survival rates following critical illness in the past decade [1, 2]. Intensive care unit (ICU) discharge no longer marks the endpoint of critical illness [3]; rather the new challenge for the 21st century is the issue of survivorship [4]. The burden of survivorship has been examined in longitudinal studies where it is evident that patients suffer ongoing muscle weakness, impaired physical functioning as well as neurocognitive and psychiatric symptoms collectively known as “post-intensive care syndrome” [1, 5–7].

Increasing numbers of patients are admitted to critical care, with this rise projected to continue as new treatments emerge, expectations for care change, and population demographics and patterns of disease alter. In ICU, patients traditionally were heavily sedated and the focus was on maintaining maximum physiological stability of the organ systems with prolonged bed rest a necessary by-product [8]. This is now being challenged as there is growing awareness that these management strategies may impact on long-term outcomes for survivors [9]. Developing a greater understanding of aetiology, mechanisms, potential treatment and preventative strategies is important for minimization of morbidity associated with survival for patients and their families including the potential economic burden on the health-care system.

### Impact of bed rest on the body systems from “bed rest” models

Bed rest was first introduced as a medical treatment in the 19th century to minimize the metabolic demand on the body and enable a focus on healing and rest to promote recovery [10]. However, lack of physical activity and prolonged bed rest have significant consequences on musculoskeletal, cardiovascular, respiratory, integumentary and cognitive systems and may be associated with harm [11]. Bed rest models are commonly used to simulate the effects of space flight and physical inactivity [12, 13]. Anti-gravity muscles such as leg extensors and trunk musculature are preferentially affected by the loss of mechanical loading compared to hand and upper limb musculature [12, 14]. Three bed rest models are commonly used for muscle wasting research.

### Limb suspension

Commonly achieved in humans using a sling and crutches (occasionally with bespoke shoes), a limb suspension model was used by de Boer et al. [15]. In a 23-day program, muscle mass was seen to reduce by 5.2 % within the first 2 weeks and subjects lost in total 10 % of quadriceps muscle mass by 23-days. Of note, this was not paralleled by continued up regulation of the intracellular signalling molecules driving muscle protein breakdown (MPB). Muscle activity across the knee joint of the suspended limb is not suppressed, suggesting that this is not the most effective model of lower limb immobilization [16].

### Limb casting

Limb casting, preventing knee joint movement, can induce further immobilization. Subjects are usually allowed to continue ambulation on crutches. Whilst numbers of subjects have been relatively small, decreases in muscle mass have been noted in several studies between 10 and 14 days [17, 18]. Gibson demonstrated decreased muscle protein synthesis (MPS) using a limb casting method, albeit over a longer period of 6 weeks [19, 20].

### Bed rest and microgravity

The most commonly used model, bed rest has been shown to cause muscle wasting within 10 days in healthy older adults [21]. However, when a head-down position is added (simulating microgravity), Ferrando et al. demonstrated loss of muscle mass within 7 days [22]. This combination has been used repeatedly to simulate muscle unloading in space flight unlike limb casting and limb suspension; bed rest ± microgravity induces the multisystem effects of immobilization [16, 22]. With immobilization, in addition to an overall reduction in muscle mass, there is a reduction in muscle fibre size [12, 14] with accelerated reduction in the strength of fast-twitch (type II) fibres compared to slow-twitch (type I) fibres which rely on oxidative metabolic processes, resulting in lower fatigue resistance capacity [12, 23]. There is not only a loss of muscle force generation capacity due to reduction in muscle mass, contractile proteins, but also alterations in muscle electromyographic activity. This highlights changes occur in terms of the neural or muscle membrane excitability to enable potentiation of a muscle contraction [24, 25]. Immobility also increases the production of pro-inflammatory cytokines and reactive oxygen species with subsequent muscle proteolysis promoting overall muscle loss [26, 27]. As a result of loss of muscle mass, up to 40 % of muscle strength can be lost within the first week of immobilization [12]. Bed rest studies have demonstrated preferential atrophy of the anti-gravity muscle groups such as soleus, back extensors and quadriceps musculature [12, 14]. Fibre atrophy may relate to the initial fibre size, which may explain partly why anti-gravity musculature which consist primarily of Type I fibres preferentially atrophy. Loss of muscle contractile protein and fibre size is only one component to the loss of force generation capability, and other factors which interplay include neural, hormonal, and cellular signalling processes.

Skeletal tissue also responds rapidly to changes in mechanical loading during bed rest [12]. There is greater bony resorption than formation, resulting in a net reduction in bone integrity and demineralization [14] which preferentially affects trabecular bone [12] and may therefore place an individual at higher risk of fractures and future morbidity and mortality. Changes in skeletal integrity occur at a slower rate compared to muscular changes, with one study reporting a 1 % reduction in bone density within the vertebral column after 1 week of immobility [28]. A recent study demonstrated early and rapid bone demineralization in individuals with acute respiratory distress syndrome and a concomitant increase in fracture risk by ~20 % [29]. This is the first study to demonstrate this within the critical care environment [29].

Other organs systems are affected too by immobilization. A full discussion on these is beyond the scope of this review. Inactivity and prolonged bed rest have also been shown to result in cardiac deconditioning affecting both the central and peripheral cardiovascular systems. Stroke volume has been shown to be reduced by 30 % within the first month of bed rest, with an associated increase in resting heart rate, and signs of orthostatic intolerance can develop within 72 h of inactivity [30–33]. These changes are mediated to a large extent by a reduction in blood volume [34]. The respiratory system is also negatively affected with development of atelectasis and increased likelihood of developing respiratory complications such as pneumonia [32]. Other secondary consequences include increased risk of thromboembolic events, pressure ulcers, insulin resistance, and development of delirium or cognitive processing impairments and alterations in sleep patterns [26, 32, 35]. While the majority of immobility related pathophysiology normalizes upon mobilization and reduction in sedation, the effects on skeletal muscle do not. Instead muscle wasting results in muscle weakness, which in critical illness is termed Intensive Care Unit-Acquired weakness (ICU-AW).

### Intensive care unit-acquired weakness—incidence and diagnosis

Intensive care unit-acquired weakness is defined as clinically detectable weakness in which there is no plausible aetiology other than critical illness [36] and is characterized by bilateral symmetrical flaccid paresis of the limbs [37]. It is associated with prolonged hospitalization, delayed weaning and increased mortality [38–40]. The reported incidence of ICU-AW varies depending on the patient population, timing of assessment and diagnostic methods used (i.e. electrophysiological, histological or clinical) [41–44]. In one prospective study, ICU-AW was clinically diagnosed in 25 % of patients receiving mechanical ventilation for greater than 7 days [41], and in another study using electrophysiological testing, the incidence was 58 % [45]. The incidence of ICU-AW is significantly higher in individuals with sepsis and has been reported to be as high as 50–100 % [46–49]. The diagnosis of ICU-AW is primarily clinical based on manual muscle strength testing using the Medical Research Council sum score to assess bilaterally six muscle groups in the upper and lower limb [50]. A score of less than 48 out of 60 is considered indicative of ICU-AW [51]. A two-tier approach to screening for the presence of ICU-AW has been recommended involving (1) evaluation of handgrip strength and (2) manual muscle strength testing using an isometric approach with the Medical Research Council sum score [52]. Additional testing can be performed such as electromyography, nerve conduction or muscle biopsy to determine the presence of neuropathy, myopathy or neuromyopathy [50]. However, these investigations are not routinely available in every clinical setting and are more time-consuming, invasive and costly to perform.

### Non-immobilization aetiology and risk factors for the development of ICU-AW

The aetiology by which critical illness leads to muscle weakness is complex and involves several inter-related processes [53]. Investigation into the risk factors for the development of ICU-AW have been limited by poor study design (predominantly retrospective chart review within a single centre), small sample sizes, lack of standardized definitions and heterogeneity of the ICU cohorts, thus limiting the comparability between studies. A number of independent risk factors have been correlated with the development of ICU-AW including sepsis [54]; the presence of organ failure involving two or more organs and severity of illness [41, 55–58]; duration of mechanical ventilation [41]; ICU length of stay [44, 54]; female gender [41]; hyperglycaemia [44, 59] and immobility [41, 53, 60]. Sepsis additionally has adverse effects on mitochondrial function, which may have a further compounding effect on muscle wasting [61]. Pathophysiologically immobility and local and systemic inflammation are believed to act synergistically to promote significant muscle loss in the critically ill patient [53]. Whilst the use of neuromuscular blockade agents has been previously cited as a risk factor, there remains no evidence for its association [62]—the only randomized trial performed showed no increase in ICU-AW following 48 h of paralysis [63], even in patients receiving concomitant steroids [64]. However, the relative contribution of each of these factors has not yet been established, and a full discussion on non-immobilization-related risk factors is beyond the scope of this review.

### Sedation—a risk factor that potentially augments the pathophysiology of immobilization

**Fig. 1:**
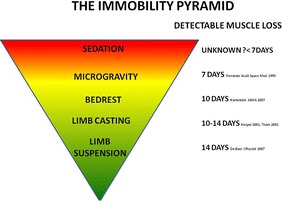
The immobility period. Providing conceptualization of the rate of muscle wasting in different states of immobilization based on the research literature

Sedation remains an unknown factor in immobilization-related muscle remodelling. Propofol and benzodiazepines positively modulate the inhibitory function of the neurotransmitter gamma-aminobutyric acid (GABA) [65, 66]. GABA facilitates the opening of the voltage-gated chloride channels in skeletal muscle, decreasing muscle excitability [66, 67]. Barbiturates and ketamine attenuate the response of excitatory neurotransmitters such as glutamate, decreasing muscle tone by acting on motor associated neurones in the spinal cord via *N*-methyl-d-aspartate (NMDA) receptors [66, 68, 69]. Recently, NMDA receptors have been discovered on the post-synaptic endplate in skeletal muscle [70]. Skeletal muscle is believed to require active support from neuronal trophic factors such as neuregulin to maintain mass. Pharmacological attenuation of their transport by sedatives may compound muscle wasting [71, 72]. Thus, continued sedation is likely to have a greater effect on muscle atrophy and weakness than “conscious immobility” in the absence of sedation (Fig. 1). It is reasonable to postulate that the beneficial effects of minimizing sedation [73–76] might, at least in part, be due to a relative maintenance of muscle mass and function. This hypothesis has yet to be formally examined. There are intrinsic difficulties in separating the effects of sedatives from bed rest in humans and ethical issues in animal studies. It may be that cell culture work, using human myocytes, is required so as to advance understanding of this issue.

### The pathophysiological effect of immobilization on skeletal muscle in critical illness

Skeletal muscle mass is regulated by a balance between MPS and MPB [77]. In a 70 kg human, approximately 280 g of protein is synthesized and degraded each day [78]. The two processes are linked, in a fashion described by Millward as facilitative or adaptive processes, whereby MPS facilitates (allows modulation of muscle mass) and MPB adapts (limiting said modulation) [79]. When exposed to an anabolic stimulus, MPS rises. MPB rises too, but to a lesser amount, resulting in a net synthetic balance. In response to an anti-anabolic stimulus, MPS decreases and MPB decreases to a lesser degree, resulting in a net breakdown.

The interaction between critical illness and bed rest may result in greater muscle loss compared to bed rest alone [26, 53]. The musculoskeletal system is a highly plastic and adaptive system, responding quickly to changing demands [43, 80, 81]. Relatively short periods of immobilization decrease MPS, with no effect on MPB [15]. Furthermore, this altered balance is relatively resistant to high dose amino acid delivery [82]. This is in contrast to studies in animals, in which MPB appears the dominant process [83, 84]. Immobilization has significant effects on peripheral muscle aerobic capacity [21], contractility [85], insulin resistance [86] and architecture [87]. Microvascular dysfunction occurring in severe sepsis is associated with immobilization and may have an additive effect on reducing MPS [86, 88]. In critically ill patients, MPS is reduced even with nutritional delivery, with increased MPB seen, leading to a net catabolic state and thus muscle wasting [58].

Muscle wasting occurs early and rapidly in the critical care setting with up to 30 % of muscle mass lost within the first 10 days of an ICU admission [58, 89]. The rate of wasting for muscle thickness was fastest for the rectus femoris muscle (9 %) compared to vastus intermedius (1 %) and vastus lateralis (0.2 %) musculature (within the quadriceps complex) in the first 72 h after an ICU admission [89]. The difference in the pattern of muscle wasting may be partly explained by the difference in functionality of the muscle and fibre type composition. Rectus femoris is a power muscle predominantly consisting of type II fibres (fast twitch) whereas vastus intermedius is a deep mono-articular stabilizer knee extensor predominantly consisting of type I fibres (slow twitch) [89].

Muscle quality or echotexture deteriorates significantly in the first 10 days of an ICU admission with infiltration of non-contractile tissue such as connective tissue and oedema [89, 90]. Changes in the muscle quantity and quality using ultrasonography have been correlated with measures of muscle strength and function [89]. Recent human studies have demonstrated a reduction in muscle myofibre size with preferential proteolysis of the thick myosin filaments [91, 92] with one trial demonstrating a dramatic increase in protein degradation of up to 160 % [92]. The same phenomenon has been observed in bed rest studies [93]. Finally, immobilization results in a loss of contractile function disproportionate to the loss of muscle mass [25]. Using single-fibre isolation methods, this phenomenon has been observed in critically ill patients [94].

### Intracellular signalling in immobilization and critical illness

The signalling pathways underlying altered protein homeostasis in immobilization has been extensively studied. Immobilization upregulates E3 ligases [95] and other components of the ubiquitin proteasome pathway [96, 97]. In critically ill patients, the same pathways are unregulated and lead to altered protein homeostasis [58]. Upstream activators of the ubiquitin proteasome pathway may exist in critical illness separate to immobilization up-regulated pathways, which may account for the differences in wasting seen between models and the clinical scenario. Local and systemic inflammatory processes in critically ill individuals may further disrupt the balance between muscle protein synthesis and protein breakdown, leading to a greater reduction in muscle mass [27, 98, 99]. Increased circulating inflammatory cytokines (such as tumor necrosis factor-alpha (TNF-α) and interleukin-1 beta (IL1-β) may also drive mitochondrial oxidative stress and increase intracellular calcium [98]. This is postulated to trigger muscle proteolytic pathways [98, 100] and interfere with insulin signalling, leading to anabolic resistance [88], and contribute to electrophysiological inexcitability of the muscle [101, 102].

Intramuscular metabolic pathway alterations have also been examined in immobilization models. Insulin resistance was first described in 1959 following bed rest in healthy subjects [103]. The development of insulin resistance subsequent to immobilization has been repeatedly observed [86, 104, 105]. Insulin resistance occurs in critical illness and immobilization may contribute to this [106]. The pathogenesis of insulin resistance seems similar—that of decrease in intramuscular glucose transporter 4 (GLUT-4) concentrations seen in immobilized muscle and in critically ill patients [106, 107]. Free reactive oxygen species and other radical species can cause upregulation of other protein degradation molecules such as calpains and caspases which can cause sarcolemma damage and interact directly with the myofilament contractile function by modifying protein structure and thus affecting contractile capacity [94, 98].

A recent paper by Files and colleagues proposed muscle dysfunction following critical illness could be categorized into early and late stages of muscle wasting [108]. The early phase occurs within days of the onset of critical illness with marked muscle protein degradation and muscle atrophy secondary to upregulation of ubiquitin–proteasome, autophagy and calpain-caspase pathways [108]. Late-phase muscle weakness refers to the ongoing impairment in muscle function due to ongoing disuse and failure to regain muscle homeostasis and pre-existing underlying neuromuscular deficits prior to ICU admission [108]. Whilst this is an attractive model to aid decision making regarding timings of exercise interventions [109], multiple barriers to early mobilization exist.

### Barriers to early activity and mobilization

Although prolonged inactivity is recognized as harmful, current levels of activity and mobilization are low from point prevalence and observational data [110–112]. The main barriers identified include sedation, the presence of endotracheal tube and potential respiratory and/or haemodynamic instability [110–112]. One study reported 47 % of barriers identified were due to modifiable factors [113]. A recent behavioural mapping study demonstrated patients in critical care are inactive outside of dedicated rehabilitation time, and individuals who were ventilated were 5 times more likely to be inactive [114]. All individuals in this study spent at least one-third of their day alone and inactive irrespective of ventilation or sedation state [114]. Low-physical activity levels and muscle strength are associated with reduced physical function at intensive care discharge [115]. Although the evidence demonstrating early rehabilitation is safe and feasible [116, 117], and the development of clinical consensus guidelines for undertaking in-bed and out of bed active mobilization [118], activity levels continue to remain low. A significant culture change is needed and staffing and environmental barriers need to be considered independently of patient-related barriers to promote early activity and mobilization.

### Potential rehabilitation strategies to reverse effects of prolonged bed rest in the ICU setting

To reverse the effects of immobilization on the musculoskeletal system, it is important to consider methods of training to enable “overload”—by placing greater demand on the muscle to potentially mitigate the severity of immobilization induced muscle wasting [24]. Considerations of the specificity of training in terms of limb position, types of training (strength, endurance, interval) as well as methods to artificially mimic physiologically induced activity are important constructs currently being investigated.

### Assistive technology

Muscle wasting occurs early and rapidly as described earlier in this review in individuals with critical illness [58]. There is growing interest in the use of assistive technologies, in particular supine cycle ergometry and muscle stimulation to commence rehabilitation early in the ICU admission without the need for direct patient engagement [119]. In a randomized controlled trial (RCT) of cycle ergometry, a significant difference was seen in the intervention cohort for 6-min walk distance and isometric quadriceps strength at hospital discharge [120]. Although this study demonstrated promising results for enhancing recovery of muscle strength and functional outcomes, there was a significant delay in the time to commencement of the intervention—2 weeks post ICU admission [120]. Artificial stimulation of the skeletal muscles through the use of low voltage electrical impulses delivered through the skin to the underlying muscle via surface electrodes [121] is another assistive modality, which can be utilized without the need for volitional activation [122]. The efficacy for electrical muscle stimulation is inconclusive [122]. Finally, there is also growing interest in functional electrical stimulation assisted cycling—stimulation of multiple muscle groups in a functional manner facilitating cycling [123]. Preliminary research has demonstrated the safety and feasibility of this form of intervention when commenced early in the ICU admission period in individuals with sepsis [123], and a large multi-centre randomized controlled trial is currently in progress to determine the efficacy in terms of functional and cognitive recovery.

### Active rehabilitation

Optimization of sedation and delirium practices is necessary to enable patient engagement with active rehabilitation. Clinical practice guidelines have been published on sedation and delirium practices with consideration of mobilization within the bundle of care [124, 125]. Schweickert and colleagues published a landmark Randomized Controlled Trial examining early physical and occupational therapy commencing within the first 48 h of ICU admission and demonstrated improved functional recovery at hospital discharge and reduced delirium duration [72]. Denehy and colleagues examined the efficacy of exercise rehabilitation commencing during the ICU admission and continuing across the continuum of recovery into the outpatient setting compared to usual care practices [46]. There was no significant difference found in terms of 6-min walk distance results at 12 months; however, exploratory analyses demonstrated the rate of change over time and mean between group differences were higher in the intervention cohort [46], and pre critical illness disease status may have been an unaccounted for factor [126]. The evidence for rehabilitation in the ICU appears to improve quality of life, physical function and muscle strength [127]; however, the optimal timing, dosage and specific intervention type have not been elucidated.

## Conclusions

Prolonged immobility is harmful with rapid reductions in muscle mass, bone mineral density and impairment in other body systems evident within the first week of bed rest, which is further exacerbated in individuals with critical illness. Therapeutic strategies to enable early rehabilitation and physical activity need to be developed alongside a culture of physical activity in the critical care setting. Addressing these concerns will enable a paradigm shift from bed rest and inactivity to physical activity and mobility in the future.

## Abbreviations

GABA: gamma-aminobutyric acid
GLUT-4: glucose transporter 4
g: grammes
kg: kilogrammes
ICU: intensive care unit
ICU-AW: intensive care unit-acquired weakness
IL-1β: interleukin-1 beta
MPB: muscle protein breakdown
MPS: muscle protein synthesis
NMDA: *N*-methyl-d-aspartate
TNFα: tumor necrosis factor-alpha
-%: percentage

## References

[1] Needham D (2012). Improving long-term outcomes after discharge from intensive care unit: report from a stakeholders’ conference. Crit Care Med.

[2] Iwashyna T, Netzer G (2012). The burdens of survivorship: an approach to thinking about long-term outcomes after critical illness. Semin Respir Crit Care Med.

[3] Needham D, Feldman D, Kho M (2011). The functional costs of ICU survivorship. Collaborating to improve post-ICU disability. Am J Respir Crit Care Med.

[4] Iwashyna T (2010). Survivorship will be the defining challenge of critical care in the 21st century—editorial. Ann Intern Med.

[5] Herridge M (2011). Functional disability 5 years after acute respiratory distress syndrome. N Engl J Med.

[6] de Rooij S (2008). Cognitive, functional, and quality-of-life outcomes of patients aged 80 and older who survived at least 1 year after planned or unplanned surgery or medical intensive care treatment. J Am Geriatr Soc.

[7] Hopkins R, Jackson J (2009). Short- and long-term cognitive outcomes in intensive care unit survivors. Clin Chest Med.

[8] Brower R (2009). Consequences of bed rest. Crit Care Med.

[9] Griffiths R, Jones C (2007). Seven lessons from 20 years of follow-up of intensive care unit survivors. Curr Opin Crit Care.

[10] Pavy-Le Traon A (2007). From space to Earth: advances in human physiology from 20 years of bed rest studies (1986–2006). EurJ Appl Physiol.

[11] Allen C, Glasziou P, Del Marc C (1999). Bed rest: a potentially harmful treatment needing more careful evaluation. Lancet.

[12] Topp R (2002). The effect of bed rest and potential of prehabilitation on patients in the intensive care unit. AACN Clin Issues.

[13] Preiser JC (2010). Effects of bedrest on muscle metabolism. Pratic Anesth Reanim.

[14] Bloomfield S (1997). Changes in musculoskeletal structure and function with prolonged bed rest. Med Sci Sports Exerc.

[15] de Boer MD (2007). The temporal responses of protein synthesis, gene expression and cell signalling in human quadriceps muscle and patellar tendon to disuse. J Physiol.

[16] Adams GR, Caiozzo VJ, Baldwin KM (2003). Skeletal muscle unweighting: spaceflight and ground-based models. J Appl Physiol.

[17] Hespel P (2001). Oral creatine supplementation facilitates the rehabilitation of disuse atrophy and alters the expression of muscle myogenic factors in humans. J Physiol.

[18] Thom JM (2001). Effect of 10-day cast immobilization on sarcoplasmic reticulum calcium regulation in humans. Acta Physiol Scand.

[19] Gibson JN (1987). Decrease in human quadriceps muscle protein turnover consequent upon leg immobilization. Clin Sci (Lond).

[20] Gibson JN, Smith K, Rennie MJ (1988). Prevention of disuse muscle atrophy by means of electrical stimulation: maintenance of protein synthesis. Lancet.

[21] Kortebein P (2008). Functional impact of 10 days of bed rest in healthy older adults. J Gerontol A Biol Sci Med Sci.

[22] Ferrando AA (1995). Magnetic resonance imaging quantitation of changes in muscle volume during 7 days of strict bed rest. Aviat Space Environ Med.

[23] Greenleaf J, Kozlowski S (1982). Physiological consequences of reduced physical activity during bed rest. Exerc Sports Sci Rev.

[24] Bruton A (2002). Muscle plasticity: response to training and detraining. Physiotherapy.

[25] Berg H, Larsson L, Tesch P (1997). Lower limb skeletal muscle function after 6 weeks of bed rest. J Appl Physiol.

[26] Winkleman C (2009). Bed rest in health and critical illness—a body systems approach. AACN Adv Crit Care.

[27] Puthucheary Z (2010). Structure to function: muscle failure in critically ill patients. J Physiol.

[28] LeBlanc A (1994). Changes in intervertebral disc cross-sectional area with bed rest and space flight. Spine.

[29] Rawal J (2015). A pilot study of change in fracture risk in patients with acute respiratory distress syndrome. Crit Care.

[30] Saltin B (1968). Response to exercise after bed rest and after training. Circulation.

[31] Convertino V (1997). Cardiovascular consequences of bed rest: effect on maximal oxygen uptake. Med Sci Sports Exerc.

[32] Convertino V, Bloomfield S, Greenleaf J (1997). An overview of the issues: physiological effects of bed rest and restricted physical activity. Med Sci Sports Exerc.

[33] Convertino V (1982). Cardiovascular responses to exercise in middle-aged men after 10 days of bed rest. Circulation.

[34] Convertino VA (1997). Cardiovascular consequences of bed rest: effect on maximal oxygen uptake. Med Sci Sports Exerc.

[35] Koo K, Fan E (2013). ICU-acquired weakness and early rehabilitation in the critically ill. JCOM.

[36] Norrenberg M, Vincent J (2000). A profile of European intensive care physiotherapists. Intensive Care Med.

[37] Stevens R (2009). A framework for diagnosing and classifying intensive care unit-acquired weakness. Crit Care Med.

[38] Ali N (2008). Acquired weakness, handgrip strength, and mortality in critically ill patients. Am J Respir Crit Care Med.

[39] De Jonghe B (2004). Does ICU-acquired paresis lengthen weaning from mechanical ventilation?. Intensive Care Med.

[40] Sharshar T (2009). Presence and severity of intensive care unit-acquired paresis at time of awakening are associated with increased intensive care unit and hospital mortality. Crit Care Med.

[41] De Jonghe B (2002). Paresis acquired in the intensive care unit: a prospective multicenter study. J Am Med Assoc.

[42] Griffiths R, Hall J (2010). Intensive care unit-acquired weakness. Crit Care Med.

[43] Puthucheary Z, Harridge S, Hart N (2010). Skeletal muscle dysfunction in critical care: wasting, weakness, and rehabilitation strategies. Crit Care Med.

[44] Witt N (1991). Peripheral nerve function in sepsis and multiple organ failure. Chest.

[45] Leitjen F (1996). Critical illness polyneuropathy in multiple organ dysfunction syndrome and weaning from the ventilator. Intensive Care Med.

[46] Denehy L (2013). Exercise rehabilitation for patients with critical illness: a randomized controlled trial with 12 months follow up. Crit Care.

[47] Connolly B (2013). Clinical predictive value of manual muscle strength testing during critical illness: an observational cohort study. Crit Care.

[48] Tennila A (2000). Early signs of critical illness polyneuropathy in ICU patients with systemic inflammatory response syndrome or sepsis. Intensive Care Med.

[49] De Jonghe B (1998). Acquired neuromuscular disorders in critically ill patients: a systematic review. Intensive Care Med.

[50] Fan E (2014). An Official American Thoracic Society clinical practice guideline: the diagnosis of intensive care unit-acquired weakness in adults. Am J Respir Crit Care Med.

[51] Hough C, Lieu B, Caldwell E (2011). Manual muscle strength testing of critically ill patients: feasibility and interobserver agreement. Crit Care.

[52] Parry S (2015). A new two-tier strength assessment approach to the diagnosis of weakness in intensive care: an observational study. Crit Care.

[53] Truong A (2009). Bench-to-bedside review: mobilizing patients in the intensive care unit—from pathophysiology to clinical trials. Crit Care.

[54] de Sèze M (2000). Critical illness polyneuropathy. A 2-year follow-up study in 19 severe cases. Eur Neurol.

[55] de Letter M (2001). Risk factors for the development of polyneuropathy and myopathy in critically ill patients. Crit Care Med.

[56] Nanas S (2008). Predisposing factors for critical illness polyneuromyopathy in a multidisciplinary intensive care unit. Acta Neurol Scand.

[57] Bednarik J (2005). Risk factors for critical illness polyneuromyopathy. J Neurol.

[58] Puthucheary Z (2013). Acute skeletal muscle wasting in critical illness. JAMA.

[59] Hermans G (2007). Impact of intensive insulin therapy on neuromuscular complications and ventilator dependency in the medical intensive care unit. Am J Respir Crit Care Med.

[60] Fan E (2008). Critical illness neuromyopathy and muscle weakness in patients in the intensive care unit. AACN Adv Crit Care.

[61] Brealey D (2002). Association between mitochondrial dysfunction and severity and outcome of septic shock. Lancet.

[62] Puthucheary Z (2012). Neuromuscular blockade and skeletal muscle weakness in critically ill patients: time to rethink the evidence?. Am J Respir Crit Care Med.

[63] Papazian L (2010). Neuromuscular blockers in early acute respiratory distress syndrome. N Engl J Med.

[64] Puthucheary Z, Hart N, Montgomery H (2010). Neuromuscular blockers and ARDS. N Engl J Med.

[65] Trapani G (2000). Propofol in anesthesia. Mechanism of action, structure-activity relationships, and drug delivery. Curr Med Chem.

[66] Rang HP, Dale MM, Ritter JM (1999). Pharmacology.

[67] Jentsch TJ (2002). Molecular structure and physiological function of chloride channels. Physiol Rev.

[68] Urazaev AK (1995). Muscle NMDA receptors regulate the resting membrane potential through NO-synthase. Physiol Res.

[69] MacDonald RL, Barker JL (1979). Enhancement of GABA-mediated postsynaptic inhibition in cultured mammalian spinal cord neurons: a common mode of anticonvulsant action. Brain Res.

[70] Malomouzh AI (2011). NMDA receptors at the endplate of rat skeletal muscles: precise postsynaptic localization. Muscle Nerve.

[71] Florini JR (1996). Stimulation of myogenic differentiation by a neuregulin, glial growth factor 2. J Biol Chem.

[72] Lebrasseur NK (2003). Regulation of neuregulin/ErbB signaling by contractile activity in skeletal muscle. Am J Physiol Cell Physiol.

[73] Strom T, Martinussen T, Toft P (2010). A protocol of no sedation for critically ill patients receiving mechanical ventilation: a randomised trial. Lancet.

[74] Schweickert WD (2009). Early physical and occupational therapy in mechanically ventilated, critically ill patients: a randomised controlled trial. Lancet.

[75] Girard TD (2008). Efficacy and safety of a paired sedation and ventilator weaning protocol for mechanically ventilated patients in intensive care (Awakening and Breathing Controlled trial): a randomised controlled trial. Lancet.

[76] Kress JP (2000). Daily interruption of sedative infusions in critically ill patients. N Engl J Med.

[77] Millward D, Wildenthal K (1980). Protein turnover in cardiac and skeletal muscle during normal growth and hypertrophy. Degradative processes in skeletal and cardiac muscle.

[78] Temparis S, Asensi M, Taillandier D, Aurousseau E, Larbaud D, Obled A, Bechet D, Ferrara M, Estrela JM, Attaix D (1994). Increased ATP-ubiquitin-dependent proteolysis in skeletal muscles of tumor-bearing rats. Cancer Res.

[79] Rennie MJ (1985). Muscle protein turnover and the wasting due to injury and disease. Br Med Bull.

[80] Flück M (2012). Regulation of protein synthesis in skeletal muscle. Dtsch Z Sportmed.

[81] Stewart C, Rittweger J (2006). Adaptive processes in skeletal muscle: molecular regulators and genetic influences. J Musculoskelet Neuronal Interact.

[82] Glover EI (2008). Immobilization induces anabolic resistance in human myofibrillar protein synthesis with low and high dose amino acid infusion. J Physiol.

[83] Caron AZ (2009). A novel hindlimb immobilization procedure for studying skeletal muscle atrophy and recovery in mouse. J Appl Physiol.

[84] Sacheck JM (2007). Rapid disuse and denervation atrophy involve transcriptional changes similar to those of muscle wasting during systemic diseases. Faseb J.

[85] Duchateau J, Hainaut K (1987). Electrical and mechanical changes in immobilized human muscle. J Appl Physiol.

[86] Hamburg NM (2007). Physical inactivity rapidly induces insulin resistance and microvascular dysfunction in healthy volunteers. Arterioscler Thromb Vasc Biol.

[87] Tomanek RJ, Lund DD (1974). Degeneration of different types of skeletal muscle fibres. II. Immobilization. J Anat.

[88] Rennie MJ (2009). Anabolic resistance in critically ill patients. Crit Care Med.

[89] Parry S (2015). Ultrasonography in the intensive care setting can be used to detect changes in the quality and quantity of muscle and is related to muscle strength and function. J Crit Care.

[90] Puthucheary Z (2015). Qualitative ultrasound in acute critical illness muscle wasting. Crit Care Med.

[91] Derde S (2012). Muscle atrophy and preferential loss of myosin in prolonged critically ill patients. Crit Care Med.

[92] Klaude M (2012). Protein metabolism and gene expression in skeletal muscle of critically ill patients with sepsis. Clin Sci.

[93] Borina E (2010). Myosin and actin content of human skeletal muscle fibers following 35 days bed rest. Scand J Med Sci Sports.

[94] Llano-Diez M (2012). Mechanisms underlying intensive care unit muscle wasting and effects of passive mechanical loading. Crit Care.

[95] Jones S (2004). Disuse atrophy and exercise rehabilitation in humans profoundly affects the expression of genes associated with the regulation of skeletal muscle mass. FASEB J.

[96] Murton A, Constantin D, Greenhaff P (2008). The involvement of the ubiquitin proteasome system in human skeletal muscle remodelling and atrophy. Biochim Biophs Acta.

[97] Sacheck J (2007). Rapid disuse and denervation atrophy involving transcriptional changes similar to those of muscle wasting during systemic diseases. FASEB J.

[98] Bloch S (2012). Molecular mechanisms of intensive care unit-acquired weakness. Eur Respir J.

[99] Khan J, Harrison T, Rich M (2008). Mechanisms of neuromuscular dysfunction in critical illness. Crit Care Clin.

[100] Shang F, Gong Z, Taylor A (1997). Activity of ubiquitin-dependent pathway in response to oxidative stress. Ubiquitin-activating enzyme is transiently upregulated. J Biol Chem.

[101] Z’Graggen W (2011). Muscle membrane dysfunction in critical illness myopathy assessed by velocity recovery cycles. Clin Neurophysiol.

[102] Z’Graggen W (2006). Nerve excitability changes in critical illness polyneuropathy. Brain.

[103] Lutwak L, Whedon G (1959). The effect of physical conditioning on glucose tolerance. Clin Res.

[104] Bergouignan A (2006). Effect of physical inactivity on the oxidation of saturated and monounsaturated dietary fatty acids: results of a randomised trial. PLoS Clin Trials.

[105] Cree M (2009). Insulin resistance, secretion and breakdown are increased 9 months following severe burn injury. Burns.

[106] Weber-Carstens S (2013). Critical illness myopathy and GLUT4 significance of insulin and muscle contraction. Am J Respir Crit Care Med.

[107] Tabata I (1999). Resistance training affects GLUT-4 content in skeletal muscle of humans after 19 days of head-down bed rest. J Appl Physiol.

[108] Files D, Sanchez M, Morris P (2015). A conceptual framework: the early and late phases of skeletal muscle dysfunction in the acute respiratory distress syndrome. Crit Care.

[109] Puthucheary Z, Hart N (2009). Intensive care unit acquired muscle weakness: when should we consider rehabilitation?. Crit Care.

[110] Berney S (2013). Intensive care unit mobility practices in Australia and New Zealand: a point prevalence study. Crit Care Resusc.

[111] Nydahl P (2014). Early mobilization of mechanically ventilated patients: a 1-day point-prevalence study in Germany. Crit Care Med.

[112] TEAM Study Investigators (2015). Early mobilization and recovery in mechanically ventilated patients in the ICU: a bi-national, multi-centre prospective cohort study. Crit Care.

[113] Leditschke I (2012). Whats are the barriers to mobilizing intensive care patients?. Cardiopulm Phys Ther J.

[114] Berney S (2015). Prospective observation of physical activity in critically ill patients who were intubated for more than 48 hours. J Crit Care.

[115] Beach L, et al. Low physical activity levels and poorer muscle strength are associated with reduced physical function at intensive care unit discharge: an observational study. Am J Respir Crit Care Med. 2014;A543.

[116] Sricharoenchai T (2014). Safety of physical therapy interventions in critically ill patients: a single-center prospective evaluation of 1110 intensive care unit admissions. J Crit Care.

[117] Adler J, Malone D (2012). Early mobilization in the intensive care unit: a systematic review. Cardiopulm Phys Ther J.

[118] Hodgson C (2014). Expert consensus and recommendations on safety criteria for active mobilization of mechanically ventilated critically ill adults. Crit Care.

[119] Needham DM, Truong AD, Fan E (2009). Technology to enhance physical rehabilitation of critically ill patients. Crit Care Med.

[120] Burtin C (2009). Early exercise in critically ill patients enhances short-term functional recovery. Crit Care Med.

[121] Maffiuletti N (2010). Physiological and methodological considerations for the use of neuromuscular electrical stimulation. Eur J Appl Physiol.

[122] Parry S (2013). Electrical muscle stimulation in the intensive care setting: a systematic review. Crit Care Med.

[123] Parry S (2014). Functional electrical stimulation with cycling in the critically ill: a pilot case-matched control study. J Crit Care.

[124] Morandi A, Brummel N, Ely E (2011). Sedation, delirium and mechanical ventilation: the ‘ABCDE’ approach. Curr Opin Crit Care.

[125] Barr J, Pandharipande PP (2013). The pain, agitation, and delirium care bundle: synergistic benefits of implementing the 2013 pain, agitation, and delirium guidelines in an integrated and interdisciplinary fashion. Crit Care Med.

[126] Puthucheary ZA, Denehy L (2015). Exercise interventions in critical illness survivors: understanding inclusion and stratification criteria. Am J Respir Crit Care Med.

[127] Kayambu G, Boots R, Paratz J (2013). Physical therapy for the critically ill in the ICU: a systematic review and meta-analysis. Crit Care Med.

